# Caloric restriction modulates the monoaminergic system and metabolic hormones in aged rats

**DOI:** 10.1038/s41598-020-76219-7

**Published:** 2020-11-09

**Authors:** Marta Portero-Tresserra, D. Rojic-Becker, C. Vega-Carbajal, G. Guillazo-Blanch, A. Vale-Martínez, M. Martí-Nicolovius

**Affiliations:** Departament de Psicobiologia i Metodologia de les Ciències de la Salut, Institut de Neurociències, Universitat Autònoma de Barcelona, Barcelona, Spain

**Keywords:** Cognitive ageing, Neural ageing

## Abstract

Caloric restriction (CR) can attenuate the general loss of health observed during aging, being one of the mechanisms involved the reduction of hormonal alteration, such as insulin and leptin. This change could also prevent age-specific fluctuations in brain monoamines, although few studies have addressed the effects of CR on peripheral hormones and central neurotransmitters exhaustively. Therefore, the variations in brain monoamine levels and some peripheral hormones were assessed here in adult 4-month old and 24-month old male Wistar rats fed ad libitum (AL) or maintained on a 30% CR diet from four months of age. Noradrenaline (NA), dopamine (DA), serotonin (5-HT) and its metabolites were measured by high-performance liquid chromatography with electrochemical detection (HPLC-ED) in nine brain regions: cerebellum, pons, midbrain, hypothalamus, thalamus, hippocampus, striatum, frontal cortex, and occipital cortex. In addition, the blood plasma levels of hormones like corticosterone, insulin and leptin were also evaluated, as were insulin-like growth factor 1 and other basal metabolic parameters using enzyme-linked immunosorbent assays (ELISAs): cholesterol, glucose, triglycerides, albumin, low-density lipoprotein, calcium and high-density lipoprotein (HDLc). CR was seen to increase the NA levels that are altered by aging in specific brain regions like the striatum, thalamus, cerebellum and hypothalamus, and the DA levels in the striatum, as well as modifying the 5-HT levels in the striatum, hypothalamus, pons and hippocampus. Moreover, the insulin, leptin, calcium and HDLc levels in the blood were restored in old animals maintained on a CR diet. These results suggest that a dietary intervention like CR may have beneficial health effects, recovering some negative effects on peripheral hormones, metabolic parameters and brain monoamine concentrations.

## Introduction

The brain undergoes a series of physiological and functional alterations as it ages, which can produce greater susceptibility to neurodegenerative disorders. The changes in the aging brain are very heterogeneous, the most noticeable alterations evident in the hippocampus, the frontal cortex and the striatum^1^. Other regions affected by ageing are the thalamus, hypothalamus, cerebellum and brainstem^2^, whereas one of the least affected regions is the occipital cortex^3^. Age-related biochemical changes include altered levels of neurotransmitters and peripheral hormones, as well as their receptor interactions. Monoaminergic neurotransmitters like DA, NA and 5-HT have been shown to decline with age in some of the aforementioned regions. In rats, DA levels diminish in the hippocampus^4–7^, striatum^5,7–11^ and brainstem^11^, while significant age-induced declines in NA have been detected in the striatum and hippocampus^6,7,9^, the midbrain^10,12^ and the pons-medulla^5^. 5-HT is also reduced in the frontal cortex, hippocampus and striatum^5–7,9,10,13^. Nevertheless, ageing appears to produce increases in the 5-hydroxyindoleacetic acid/serotonin ratio (5-HIAA/5-HT) in the frontal cortex, amygdala and striatum, suggesting an age-related decrease in 5-HT synthesis is coupled to its enhanced metabolism^5^.

Age-related impairment of brain function is also associated with other physiological changes, including alterations of peripheral hormones like insulin^14^, leptin^15^ and insulin-likegrowth factor 1 (IGF-1)^16^, as well as increased levels of stress hormones like corticosterone^17^. Dysregulation of insulin and leptin affects neuronal inflammation and oxidative stress, as reflected by the changes in proinflammatory cytokine release^18,19^. In this context, it was proposed that immune system pathways may influence the biosynthesis of monoamines^20^ and indeed, age-related brain inflammation has been associated with abnormal tryptophan and tyrosine metabolism^21^, which may in turn be associated with decreases in the synthesis of monoamines during aging.

The rate and severity of the deleterious effects of normal aging can be attenuated by a wide variety of factors, such as appropriate diets. CR, defined as a reduction in the caloric intake without causing malnutrition^22^, ameliorates brain aging by reducing oxidative stress, improving mitochondrial function, activating anti-inflammatory responses, promoting neurogenesis and enhancing synaptic plasticity^23,24^. In addition, CR seems to modify leptin and insulin concentrations and metabolic parameters, which in turn may attenuate the age-related decreases in monoamines. Despite the relationship between CR, monoamines and peripheral hormones, no studies have yet combined and correlated these elements, analyzing their relationships in different brain areas.

In this context, the present study set out to evaluate the effect of life-long CR on monoamines in 24-month old Wistar rats, evaluating NA, DA and 5HT, as well as their metabolites L-DOPA, 3,4-dihydroxyphenylacetic acid (DOPAC), tyrosine hydroxylase (TH), homovanilic acid (HVA), 5-HIAA, tryptophan hydroxylase (TrpH), and the peripheral hormones insulin, leptin, corticosterone, IGF-1. Monoamines were analyzed in the brain regions most affected by aging, such as the prefrontal cortex, hippocampus, striatum and thalamus, as well as in the cerebellum, midbrain and pons, using the occipital cortex as a control region. To control the effects of CR on the animals’ general health, other biochemical parameters were also analyzed, such as their cholesterol, glucose, total protein, triglyceride, albumin, calcium, low-density lipoprotein (LDL-c), high-density lipoprotein (HDL-c) and alkaline phosphatase (ALP).

## Results

### The effects of CR on monoaminergic systems

#### Noradrenergic system

The concentration of neurotransmitters and their metabolites was assessed in each brain region (mean ± SEM, ng/g tissue: Table 1) and in general, the concentration of monoamines differed between old CR and AL animals. ANOVA revealed significant between-group differences in the NA levels in the cerebellum [F_(2,27)_ = 4.341; p < 0.05], midbrain [F_(2,27)_ = 5.844; p < 0.01], hypothalamus [F_(2,27)_ = 4.695; p < 0.05], thalamus [F_(2,27)_ = 3.60; p < 0.05], hippocampus [F_(2,27)_ = 4.92; p < 0.05] and striatum [F_(2,27)_ = 5.612; p < 0.01]. The contrast analysis identified a decrease in NA in aged animals relative to the adult group in the cerebellum, midbrain, hypothalamus and striatum, and an increase in the hippocampus. Nevertheless, the CR intervention compensated such deficits as old CR animals had higher levels of NA than old AL rats in the cerebellum, hypothalamus, thalamus and striatum, with levels similar to those in the adult animals.

**Table 1:** The concentration of brain monoamines and their metabolites (mean ± SEM; ng/g tissue) in each brain area isolated from adult rats, old rats fed Ad libitum (AL) or under caloric restriction (CR).

	Adult	Old AL	Old CR
**Noradrenaline**			
Cerebellum	179.06 ± 15.66	96.20 ± 31.93^#^	178.11 ± 18.37*
Pons	412.86 ± 23.38	415.61 ± 58.67	434.26 ± 28.30
Midbrain	629.29 ± 47.76	428.07 ± 51.74^##^	523.36 ± 22.20
Hypothalamus	1328.70 ± 191.82	751.01 ± 86.59^#^	1423.97 ± 153.20*
Thalamus	498.87 ± 47.99	419.22 ± 59.87	592.32 ± 33.26*
Hippocampus	268.87 ± 22.15	395.11 ± 48.66^#^	375.41 ± 22.67
Striatum	976.39 ± 134.14	498.96 ± 130.78^#^	1002.17 ± 79.79*
Prefrontal cortex	476.17 ± 48.41	390.69 ± 63.93	476.83 ± 31.68
Occipital cortex	203.17 ± 20.61	267.95 ± 41.51	257.31 ± 22.64
**L-Dopa**			
Cerebellum	20.21 ± 3.51	56.16 ± 32.28	47.67 ± 20.71
Pons	N.D	N.D	N.D
Midbrain	58.14 ± 31.60	18.84 ± 06.52	N.D
Hypothalamus	40.97 ± 13.51	16.13 ± 03.72	176.41 ± 56.84
Thalamus	20.61 ± 13.01	19.41 ± 12.73	28.94 ± 10.76
Hippocampus	N.D	06.66 ± 03.21	09.42 ± 06.72
Striatum	330.93 ± 77.09	179.15 ± 96.29	183.96 ± 57.47
Prefrontal Cortex	61.33 ± 6.96	55.41 ± 04.31	58.74 ± 11.64
Occipital Cortex	2.42 ± 14.16	09.96 ± 02.11	05.00 ± 01.42
**Dopamine**			
Cerebellum	N.D	N.D	N.D
Pons	134.51 ± 47.68	77.47 ± 22.00	98.99 ± 14.25
Midbrain	255.00 ± 33.49	200.41 ± 20.85	283.86 ± 14.18
Hypothalamus	318.89 ± 46.95	235.70 ± 36.23	411.29 ± 46.65* 46.65*
Thalamus	300.67 ± 83.55	268.38 ± 51.20	320.25 ± 47.24
Hippocampus	11.16 ± 07.68	40.89 ± 15.55	35.55 ± 17.26
Striatum	4178.41 ± 385.29	2129.84 ± 508.70^##^	4196.11 ± 144.25**
Prefrontal cortex	1268.12 ± 134.00	1176.80 ± 212.47	1077.88 ± 115.95
Occipital cortex	187.15 ± 36.63	373.72 ± 137.95	189.56 ± 16.33
**DOPAC**			
Cerebellum	100.21 ± 28.08	100.21 ± 28.08	100.21 ± 28.08
Pons	22.97 ± 12.35	20.70 ± 16.96	74.93 ± 15.38
Midbrain	255.18 ± 21.47	166.19 ± 32.96^#^	246.17 ± 12.73*
Hypothalamus	298.11 ± 49.03	229.81 ± 35.14	345.52 ± 43.31
Thalamus	377.47 ± 98.38	275.45 ± 76.68	436.01 ± 88.43
Hippocampus	115.34 ± 18.44	352.29 ± 103.33^#^	178.25 ± 20.44
Striatum	4268.77 ± 123.27	2779.46 ± 573.78^##^	4245.95 ± 144.49 **
Prefrontal cortex	1288.30 ± 59.04	832.73 ± 15.99^##^	1104.55 ± 57.98
Occipital cortex	212.29 ± 26.07	02.33 ± 48.89	212.32 ± 01.78
**Homovanillic acid**			
Cerebellum	3.95 ± 0.10	155.78 ± 92.56	17.08 ± 08.97
Pons	7.61 ± 03.99	27.09 ± 19.40	71.28 ± 13.52
Midbrain	122.25 ± 17.92	114.67 ± 21.37	87.43 ± 09.89
Hypothalamus	110.44 ± 20.58	109.77 ± 19.71	121.10 ± 25.71
Thalamus	142.31 ± 23.83	98.48 ± 28.25	139.41 ± 19.92
Hippocampus	34.76 ± 14.41	117.21 ± 51.75	16.96 ± 09.74*
Striatum	1246.33 ± 80.05	797.07 ± 162.56^#^	998.26 ± 56.30
Prefrontal cortex	382.27 ± 16.43	311.23 ± 47.48	307.82 ± 13.37
Occipital cortex	109.80 ± 16.79	122.49 ± 29.51	95.07 ± 04.18
**Serotonin**			
Cerebellum	78.60 ± 32.29	76.29 ± 37.26	147.48 ± 26.99
Pons	453.59 ± 29.11	253.73 ± 77.54^##^	503.08 ± 14.88**
Midbrain	936.83 ± 118.37	503.97 ± 32.49^##^	702.49 ± 39.84
Hypothalamus	626.52 ± 116.54	298.27 ± 57.03	729.82 ± 89.51*
Thalamus	395.18 ± 37.19	326.46 ± 31.48	446.43 ± 41.55
Hippocampus	372.07 ± 23.74	495.17 ± 55.82^#^	336.96 ± 20.13**
Striatum	341.67 ± 46.99	136.46 ± 31.77^##^	353.22 ± 27.06**
Prefrontal cortex	405.08 ± 41.65	403.83 ± 35.60	488.08 ± 23.26
Occipital cortex	335.18 ± 35.23	273.21 ± 52.45	274.01 ± 15.27
**5-Hydroxy-indolacetic-acid**			
Cerebellum	214.40 ± 20.58	269.45 ± 44.54	178.21 ± 15.77
Pons	434.22 ± 25.32	646.52 ± 92.12^#^	603.46 ± 37.59
Midbrain	1062.85 ± 82.62	932.61 ± 130.99	918.71 ± 38.87
Hypothalamus	1033.65 ± 158.19	816.06 ± 123.16	1241.71 ± 136.36
Thalamus	799.05 ± 72.42	600.92 ± 75.92	933.99 ± 49.82
Hippocampus	626.54 ± 33.02	985.22 ± 113.10^##^	630.18 ± 31.30**
Striatum	669.93 ± 55.40	594.11 ± 81.58	679.15 ± 42.59
Prefrontal cortex	622.34 ± 25.28	779.39 ± 147.68	633.71 ± 19.26
Occipital cortex	446.23 ± 40.99	520.49 ± 61.44	412.28 ± 15.46

Post-hoc comparisons between Old AL and Old CR (*p < 0.05; **p < 0.01) and between Adult and Old AL (^#^*p* < 0.05; ^##^*p* < 0.01) groups: *N.D.* no detectable.

#### Dopaminergic system

**Figure 1:**
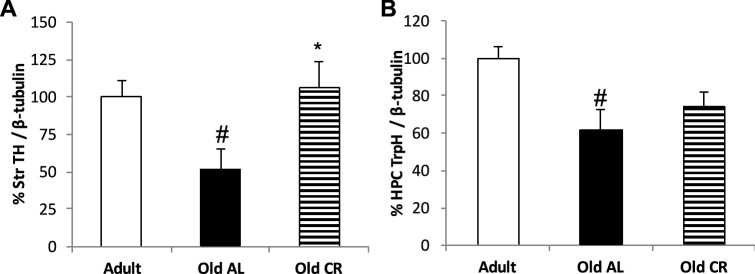
Histograms of the integrated densitometry of proteins represented as the percentage change, taking the intensity in adult’ rats as 100% for each region: (**A**) Striatum TH and (**B**) Hippocampal TrpH. The values represent the mean ± SEM of the three groups: ^#^p < 0.05 Old Al vs Adult; *p < 0.05 Old AL vs Old CR.

Some significant between-group differences in the dopaminergic system were detected in specific brain areas (Table 1). There was a significant decrease in DA in the striatum of old AL animals relative to adult animals [F(2,27) = 10.966, *p* < 0.001], as well as in DOPAC [F(2,27) = 7.659, *p* < 0.01], HVA [F(2,27) = 4.987, *p* < 0.05] and TH [F(2,17) = 4.248, *p* < 0.05] (Fig. 1A,B and Fig. 1 supplementary information), such a decline was attenuated in the old CR group. Moreover, old AL rats had less DOPAC in the midbrain [F(2,27) = 4.252, *p* < 0.05] and prefrontal cortex [F(2,27) = 6.514, *p* < 0.01], and an increase was seen in the hippocampus [F(2,27) = 5.183, *p* < 0.05]. The old CR group had higher levels of DOPAC in the midbrain and more DA in the hypothalamus [F(2,27) = 3.733, *p* < 0.05] than the old AL rats, with similar levels to the adult group. There were no significant differences in L-DOPA in any of the brain areas studied.

#### Serotoninergic system

There were significant between-group differences in the levels of 5-HT in the midbrain [F(2,27) = 7.461, *p* < 0.01], hypothalamus [F(2,27) = 4.695, *p* < 0.05] and Striatum [F(2,27) = 10.096, *p* < 0.001], and in the levels of 5-HT and 5-HIAA in the Pons [F(2,27) = 9.650, *p* < 0.001; F(2,27) = 4.516, *p* < 0.05]. In the hippocampus, there were also significant between-group differences in 5-HT [F(2,27) = 6.077, *p* < 0.01], 5-HIAA [F(2,27) = 10.677, *p* < 0.001] and TrpH [F(2,18) = 5.170, *p* < 0.05] (Fig. 1C,D). The contrast analysis showed that the old AL animals expressed less 5-HT in the pons, midbrain and striatum than the adult rats. Moreover, old CR animals had more 5-HT in the pons, hypothalamus and striatum than those in the old AL group. Additionally, in old AL animals there was more 5-HIAA in the pons and hippocampus than in adult rats, while an increase in 5-HIAA in the thalamus of old CR animals was accompanied by a decrease in the hippocampus relative to old AL.

### The effects of CR on peripheral hormones

In terms of the peripheral hormones evaluated, there was a significant between-group difference in insulin [F(2,28) = 5.843, *p* = 0.008] and leptin [F(2,28) = 21.821, *p* < 0.001] (Table 2), while no such differences were detected in corticosterone [F(2,28) = 0.688, *p* = 0.511] and IGF-1 [F(2,28) = 0.481, *p* = 0.624]. Contrast analysis indicated that the old AL animals exhibited significantly more insulin and leptin than adult rats, which was partially ameliorated in the old CR group. However, no significant differences were detected between old CR and adult rats.

**Table 2:** Concentration of blood plasma hormones in adult rats, old rats fed Ad libitum (AL) or under caloric restriction (CR).

	Adult	Old AL	Old CR	ANOVA
Corticosterone (ng/mL)	29.02 ± 5.54	41.39 ± 11.73	36.11 ± 4.39	F_(2,28)_ = 0.688 p = 0.511
Insulin (µg/mL)	0.41 ± 0.18	1.29 ± 0.18 ^**##**^	0.62 ± 0.17*	F_(2,28)_ = 5.843 p = 0.008
Leptin (pg/mL)	2111.95 ± 437.81	15,365.98 ± 2203.27^**###**^	5417.77 ± 1211.08***	F_(2,28)_ = 21.82 p < 0.001
IGF-I (pg/mL)	1,067,865.39 ± 76,210.95	977,539.08 ± 70,477.97	983,624.79 ± 65,631.44	F_(2,28)_ = 0.481 p = 0.624

Post-hoc comparisons between old AL and old CR rats (*p < 0.05; **p < 0.01; ***p < 0.001) and between adult and old AL rats (^#^p < 0.05; ^##^p < 0.01; ^###^p < 0.001) (F = Fisher-Snedecor statistical value).

### Correlations between hormone and monoamine levels

When the relationship between the hormones and brain monoamine levels were assessed, significant differences were evident (Table 3). The levels of both insulin and leptin were negatively correlated with the levels of NA in the striatum and prefrontal cortex (p < 0.05), and with the DOPAC, DA and 5-HT levels in the striatum (p < 0.05). Moreover, leptin was negatively correlated to the levels of DOPAC in the prefrontal cortex (p < 0.05).

**Table 3:** One-tailed Spearman correlation of the insulin and leptin levels with the NA and DOPAC in the striatum and prefrontal cortex, and with the levels of dopamine and serotonin in the striatum (* p < 0.05; ** p < 0.01).

	NA Striatum	NA Prefrontal	DOPAC Striatum	DOPAC Prefrontal	DA Striatum	5-HT Striatum
**Insulin**						
Spearman	− 0.437	− 0.341	− 0.529		− 0.479	− 0.465
Sig	0.016*	0.038*	0.002**		0.005**	0.006**
**Leptin**						
Spearman	− 0.400	− .0396	− 0.443	− 0.359	− 0.532	− 0.535
Sig	0.017*	0.018*	0.009**	0.030*	0.002**	0.002**

### The effects of CR on biochemical parameters

Regarding the other biochemical parameters assessed, significant between-group differences were only detected for the levels of triglyceride [F_(2.28)_ = 8.479. p = 0.001], albumin [F_(2.28)_ = 7.836. p = 0.002], calcium [F_(2.28)_ = 4.833. p = 0.016] and HDL-c [F_(2.28)_ = 3.841. p = 0.035], but not for the other parameters examined: glucose F_(2.28)_ = 1.961, p = 0.161; total protein F_(2.28)_ = 2.458, p = 0.105; ALP F_(2.28)_ = 3.008, p = 0.067; cholesterol F_(2.28)_ = 1.516, p = 0.241; and LDL-c F_(2.28)_ = 3.012, p = 0.067 (Table 4). A post hoc analysis demonstrated that the old AL group accumulated more triglycerides (p < 0.01) and less albumin (p < 0.05), calcium (p < 0.05) and HDL-c (p < 0.05) than the adult rats. Moreover, the old CR rats had more albumin (p < 0.01), calcium (p < 0.05) and HDL-c (p < 0.05) than the old AL rats. In terms of the other parameters measured, no significant differences were detected between the old CR and adult rats.

**Table 4:** Concentration of metabolic blood plasma parameters in adult rats, and old rats fed Ad libitum (AL) or under caloric restriction (CR).

	Adult	Old AL	Old CR	ANOVA
Glucose (mg/dL)	116.80 ± 3.64	147.96 ± 20.72	131.54 ± 4.15	F_(2,28)_ = 1.961 p = 0.161
Total protein (g/dL)	6.61 ± 0.10	6.55 ± 0.23	6.95 ± 0.10	F_(2,28)_ = 2.458 p = 0.105
Triglyceride (mg/dL)	80.12 ± 6.37	124.46 ± 9.55^##^	111.30 ± 6.68	F_(2,28)_ = 8.479 p = 0.001
Albumin (g/dL)	3.21 ± 0.05	2.92 ± 0.18^#^	3.46 ± 0.05**	F_(2,28)_ = 7.836 p = 0.002
Calcium (mg/dL)	10.07 ± 0.08	7.39 ± 1.58^#^	10.60 ± 0.15*	F_(2,28)_ = 4.833 p = 0.016
ALP (UI/L)	135.50 ± 17.81	77.27 ± 24.69	129.02 ± 11.81	F_(2,28)_ = 3.008 p = 0.067
Cholesterol (mg/dL)	104.13 ± 4.72	101.53 ± 11.03	121.40 ± 9.93	F_(2,28)_ = 1.516 p = 0.241
LDL-c (mmol/L)	0.45 ± 0.04	0.62 ± 0.08	0.66 ± 0.07	F_(2,28)_ = 3.012 p = 0.067
HDL-c (mmol/L)	1.3444 ± 0.05	1.0738 ± 0.13^#^	1.4083 ± 0.07*	F_(2,28)_ = 3.841 p = 0.035

Post-hoc comparisons between old AL and CR rats (*p < 0.05; **p < 0.01; ***p < 0.001), and between adult and old AL rats (^#^p < 0.05; ^##^p < 0.01; ^###^p < 0.001). (F = Fisher-Snedecor statistical value).

## Discussion

In this study, maintaining a CR diet from 4-months of age ameliorated the decline in monoamines in specific brain areas, as well as the increase in plasma leptin and insulin associated with aging. The correlation results also indicate that the beneficial effects of CR on some peripheral hormones parallel the positive changes in monoamine neurotransmission in old animals. Moreover, the analysis of several biochemical parameters, such as glucose, total protein and ALP, did not indicate health alterations associated with the CR diet.

Our findings suggest that the changes in monoamines and the effects of the CR diet might be specific for particular brain regions and specific neurotransmitters. Thus, there is a decrease in the NA in the striatum, cerebellum and midbrain of old AL rats, and an increase in the hippocampus relative to the adult rats. These results are consistent with previous data in rats whereby age-related deficits in noradrenergic transmission were described in the midbrain^5,10,12^ and striatum in male^7^ and in female^6^ animals. Moreover, an increase of NA in the hippocampus has also been reported previously^10,12^. Although aging has also been seen to produce a decrease in hippocampal NA in old male Wistar rats^9^ and female Long-Evans rats^6^ or even no changes^7,8^, as in the hypothalamus^7,25,26^ and frontal cortex in male^8^ and in females rats^6^. Interestingly, CR increased NA in these brain regions relative to rats fed AL, as well as in the thalamus and cerebellum. Moreover, previous studies indicated increases in NA levels in the hippocampus^27^ and decreases in the caudate nucleus and hypothalamus^28^. In the latter study, in which CR diet was more severe than the one used in the present study, the NA reduction in the striatum was detected in male but not in female rats, contrary to what was observed in the hypothalamus.

The results presented here confirm that the levels of DA and its metabolites tend to decline with age in the striatum of male Wistar rats^5,8–10,13,29^. However, results in female rats seem to be different, as no changes of DA in this brain area have been reported^6,30^. Indeed, a previous study reported that neurons of the *substantia nigra* degenerate with age in male rats, especially those projecting to the dorsolateral region of the striatum^31^. In turn, a lower rate of DA replacement^25^, and in the synthesis and expression of the DA transporter, has been seen in the striatum of male aged rats^32^. Regarding the hippocampus, no changes in the levels of DA and metabolites have been detected, similarly to previous reports in male rats^4,7,8,13,30,33^, but in contrast to the decreases observed in Long-Evans female rats^6^ and Sprague-Dawley male rats^4,5^. In terms of the effects of diet and when compared to old AL animals, old rats subjected to CR expressed more TH, DOPAC and DA in the striatum, more DOPAC in the frontal cortex and midbrain, and less DOPAC in the hippocampus. Previous studies in male rats have found that CR increased DA in the *Substantia nigra*^34,35^, and that a reduction in body weight improved DA receptor signaling^36^ and enhanced striatum DA release^37^. However, a more severe CR diet reduced DA in the striatum of male rats^28^.

Concerning the serotonergic system, the old AL rats exhibited more 5-HT and 5-HIAA in the hippocampus than adult animals, a modification that was reverted in the CR rats. Increases in HT and/or 5-HIAA in the hippocampus have been previously reported in rats^4,5,7,8,29^ but such a results are in disagreement with others who found no decreases^9,10,30^ or even no effect of age^6,11,13,33^. An age-dependent decline in the serotoninergic pathways was also found here in the striatum^5–7,9,13^ and hypothalamus^26^. In addition, CR recovered the age-dependent 5-HT decrease in the pons^29^, midbrain, hypothalamus and striatum. In general, CR seems to combat the effects of aging on monoamine levels, although the action of CR in specific brain areas remain unclear as the type of diet, age, sex and strain/species may affect, suggesting that neurotransmitter brain levels are highly sensitive to such variables that are important to take into consideration in future studies. In fact, it has been described that dietary interventions can have different effects on monoamines depending on the sex of the animals^6,7,30^.

The effects of CR on peripheral hormones like leptin and insulin are consistent with earlier studies confirming that CR attenuates the age-related increase in insulin and leptin^38^. Indeed, the reduced activity of these metabolic pathways promotes health and enhances the animal’s lifespan^39^. Moreover, both insulin and leptin are negatively correlated with the levels of NA in the striatum and prefrontal cortex, and the levels of DOPAC, DA and 5-HT in the striatum, while the leptin concentration is also negatively correlated to DOPAC levels in the prefrontal cortex, which suggests that regulating these hormones through CR may enhance monoaminergic neurotransmission in specific brain regions. As for other hormones, no differences in IGF-1 and corticosterone were evident between adult and old animals, which contrasts with earlier studies showing that dietary interventions might diminish^40^ or even augment^16^ serum IGF-1 levels, and upregulate circulating blood levels of corticosterone^41^. However, similar levels of corticosterone were previously found in aged AL and CR animals^42^, in accordance with our results and confirming that CR does not affect stress hormones.

To control the effects of CR on the animals’ general health, biochemical parameters were analyzed, such as cholesterol, glucose, total proteins, triglycerides, albumin, calcium, LDL-c, HDL-c and ALP. Old AL rats had more triglycerides and less HDL-c, albumin and calcium in the blood than the adult rats, while CR reverted some of these changes (e.g. triglycerides, albumin, HDL-c and calcium in the blood). The LDL-c levels were higher in both the aged groups relative to the adult animals, in agreement with a previous study on the effects of dietary restriction in rats^43^. Moreover, our results confirm the capacity of CR to attenuate triglycerides, thereby improving cardiac function and health during aging^44^. Finally, no changes in total protein and glucose have been detected due to aging, although CR could ameliorate the age related decline in plasma albumin and calcium.

The present study is one of the few experimental studies to investigate the effects of CR on monoamines and peripheral hormones, although some limitations should be noted. First, the experiment was performed on males and as discussed before, sex may be an important variable to consider. Another important factor is the experimental design, as we used a long-life dietary intervention evaluating the animals when they were 24-months-old, while other studies have used shorter periods of CR or intermittent interventions. Moreover, we did not record the biometric parameters of animals, such as body fat, lean body mass, bone mass or length. Considering that the CR lasted for almost two years, these parameters could well be of particular interest to assess the effects of CR on the animals’ health. Furthermore, the lack of cognitive data makes it difficult to interpret the functional effects associated with the neurochemical changes found. However, other studies in our laboratory showed positive effects of CR on monoaminergic transmission that correlate with better spatial memory^27^. Thus, further research is needed to identify the potential role of sex-dependent differences in caloric requirements and how a CR diet applied at different time points in life affects monoamine levels^5,13^.

Currently, dietary interventions and healthier eating habits remain the most applicable and cost-efficient means of preventing a wide variety of age-related diseases. Although a low-caloric diet throughout life is unlikely to be feasible for most people, a growing body of evidence demonstrates that CR exerts beneficial effects on brain aging at multiple levels. In a recent study on humans^45^, it was demonstrated that reduced caloric intake is geroprotective, which indicates that this metabolic intervention influences the immune system and modifies the aging process. However, further studies will be needed to fully elucidate whether CR may represent a novel therapeutic intervention for healthy aging.

In summary, it has been long known that CR has some protective effects on aging brain, but studies investigating the biochemical effects of CR on brain aging are scarce. In this study, we show that in general, a lifelong CR diet can attenuate the age-related decline in monoaminergic neurotransmission observed in some brain regions. The beneficial effects of CR on brain monoamines parallels the improved metabolic regulation. Hence, it cannot be ruled out that attenuation of the age-related increase in insulin and leptin may help to restore brain monoamine levels in brain areas like the hippocampus and frontal cortex.

## Materials and methods

### Subjects

This study was carried out on 30 male Wistar rats from our laboratory’s breeding stock (Prolabor, Charles River Laboratories, Abresle, France). The CR group of old rats (n = 12; age = 23–24 months; weight = 483.1 g ± 91 g) were kept under conditions of CR from four months of age, with a 30% reduction of total food intake (18–20 g/day) coupled to free access to water. Another group of old rats (n = 8; age = 23–24 months; weight = 789.1 g ± 68.2 g) were given Ad libitum (AL) access to food and water, as was a control group of adult animals (n = 10; age = 3–4 months; weight = 369.6 ± 22.9 g). The animals were fed with dry pellets (Harlan Laboratories Inc., Madison, USA) produced and packed by Mucedola Sri. (MI, Italy). Animals were pair-housed from the beginning of the experiment and maintained at a constant temperature (21 °C) on a controlled 12 h light/dark cycle. The animal housing procedures are explained in more detail elsewhere^46^. All procedures were performed in compliance with protocols approved by the Animal Care and Use Committee of Autonomous University of Barcelona (CEEAH number 3866), with authorization from Department of Environment of the Generalitat de Catalunya, and with guidelines approved by the EU Council Directive for the care and use of laboratory animals (2010/63/EU).

### Blood samples and plasma analysis

Food was removed the day before sacrifice to minimize the biochemical and hormonal differences between the groups due to the amount of food consumed immediately before^47^ or due to lipidemia^48^. After sacrificing the animals by decapitation, blood samples were collected in Heparin tubes (Sodium Heparin, 5000 USP/mL; Chiesi Spain, SA, Spain) and placed on ice for slow coagulation. Cell-free plasma was obtained by centrifugation at 6000 rpm for 15 min at 4 °C (RCF 2361 × *g*) and it was stored at − 80 °C until biochemical analysis. These frozen plasma samples were allowed to reach room temperature before processing and analyzing the levels of: corticosterone, insulin, leptin, IGF-1, cholesterol, glucose, total protein, triglycerides, albumin, LDL-c, calcium, HDL-c and ALP. Corticosterone levels were measured at the *Servei de Bioquímica, Clinica Veternitaria* (Universitat Autònoma de Barcelona) with a competitive Corticosterone EIA (Immunodiagnostic Systems Ltd, Bolton, UK), while the insulin, leptin and IGF-1 levels were assessed using Sandwich ELISAs, all quantified on a EMS Reader MF V.2.9-0: Mercodia Rat Insulin ELISA (Mercodia AB, Sweden); Quantikine ELISA Mouse/Rat leptin (R&D Systems, Inc. USA) Quantikine Mouse/Rat IGF-I ELISA (R&D Systems, Inc. USA). Cholesterol was analyzed by the CHE/POD1 enzyme method, glucose by the Hexokinase method, and the total protein was calculated by the Biuret method. Triglycerides were quantified by the Glycerol-3-phosphate oxidase method, albumin levels by the Bromocresol green method, calcium levels by the Arzenazo III method, LDL-c was quantified by the selective protection method and HDL by the immunoinhibition method, and the ALP levels were assessed by the Substrate 4-nitrophenyl phosphate method using AMP buffer. All these analyses were carried out on an AU400 Olympus analyzer (Germany) with OSR reagents (Olympus System Reagent, Beckman Coulter, Ireland).

### Tissue collection and processing

After decapitation, the rat’s brain was rapidly removed and dissected into different regions that were weighed, frozen and stored at − 80 °C. The following bilateral brain regions were collected and processed for HPLC-ED analysis: frontal cortex, hippocampus, striatum, thalamus, cerebellum, pons, midbrain, hypothalamus, and occipital cortex. The striatum and hippocampus were also analyzed in Western Blots.

### Quantification of monoamines and their metabolites by HPLC-ED

Brain samples were homogenized in buffer (perchloric acid 60% w/w 0.25 M, sodium metabisulphite 100 µM, EDTA Na_2_·2H_2_O 250 µM) in a 9/1 ratio (p/v; ml/mg). A polytron homogenizer was used to rapidly disrupt the animal tissue and the homogenate was centrifuged for 10 min at 15,000 rpm and 4 °C (5417 R centrifuge). The supernatant recovered was filtered and 50 µL aliquots were analyzed on a reverse phase column (Cromolith Performance, 4.6 mm internal diameter × 10 cm length) coupled to a pre-column (4.6 mm × 5 cm). The mobile phase consisted of 0.1 M citric acid, 0.05 M EDTA, 1.2 mM SOS, 10% acetonitrile (v/v) adjusted to pH 2.75 with tetraethylammonium. Elution was performed at a flow rate of 0.8 mL/min and the HPLC apparatus (LaChrom Elite) was coupled to an electrochemical detector (ED: ESA Coulochem 5100A), with an ESA analytical dual electrode cell 5011A (the detection potential for electrodes 1 and 2 was set at 70.05 and + 0.4 V, respectively). Before and after each round of processing the samples, one calibration mix was analyzed at seven dilutions: 12.5, 25, 50, 62.5, 125, 250 and 500 μg/mL. Depending on the retention time of each component, calibration lines (r^2^ = 0.999) allowed of the concentration of the monoamines (NA, DA, 5-HT) and their metabolites (L-DOPA, DOPAC, HVA, 5-HIAA) to be calculated. EZChrom Elite Software was used to determine the appropriate concentrations, expressed as ng/g tissue.

### Semi-quantitative western blots

Striatal and hippocampal tissue was collected at different time-points and washed in lysis buffer (25 mM Tris–HCl, 150 mM NaCl, 0.5% Sodium deoxycholate, 0.1% SDS, 1% NP-40 [pH 7.6]). The lysates were homogenized with a pestle (Sigma-Aldrich Corp., Madrid, Spain), sonicated and quantified using a BCA assay (Pierce Chemical Co.). Equal amounts of protein (30 µg/well) were resolved on SDS-PAGE gels and transferred at 100 V to a nitrocellulose membrane for 1 h (Whatman, Dassel, Germany) in a Mini TransBlot Cell (Bio-Rad; Hercules, CA, USA). The membranes were blocked for 1 h at 20–25 °C with 5% non-fat dry milk in Tris-buffered saline (TBS: 75 mM NaCl, 1.5 mM KCl, 12.4 mM Tris [pH 7.4]) and they were then probed overnight at 4 °C with the corresponding primary antibody diluted in 5% (w/v) bovine serum albumin (BSA), with shaking. The following primary antibodies were used: monoclonal mouse anti-tyrosine hydroxylase (T2928, Sigma, USA, 1:1000), monoclonal mouse anti-tryptophan hydroxylase (T06981, Sigma, USA, 1:500), and mouse anti-β-tubulin (Becton–Dickinson, Franklin Lakes, NJ, USA, 1:1000). After several washes with TBS 0.1% + Tween 20, the membranes were incubated for 1 h with the corresponding horseradish peroxidase conjugated secondary antibody, anti-mouse-HRP (Dako Denmark, Glostrup, Denmark, 1:3000). Blots were developed using a chemoluminiscent mix 1:1 (0.5 M luminol, 79.2 mM p-coumaric acid, 1 M Tris–HCl [pH 8.5], and 8.8 M hydrogen peroxide, 1 M Tris–HCl [pH 8.5]). All the samples to be compared were processed at the same time, transferred simultaneously to a membrane and probed with the same antibody dilution. The apparent molecular weight of the proteins was determined by calibrating the blots with pre-stained molecular weight markers (All Blue: Pierce Chemical Co., USA) and densitometry was carried out using ImageJ software (National Institute of Health, Bethesda, MD, USA). The total content of each specific protein (TH and TrpH) was assayed after the membranes were stripped for 1 h at 20–25 °C with Glycine (0.1 mM, pH 2.3), blocked again and incubated with the corresponding primary antibody. Chemiluminescence signals of the bands obtained were all within the linear range of the imaging system and were not saturated (ChemiDoc XRS + System, Bio-Rad Laboratories). Densitometry and quantitation was carried out using ChemiDoc MP Imaging System, Image Lab program (Bio-Rad) and Microsoft Excel was used to determine the levels of proteins. The adult group of subjects was used as a control group, and the total level of proteins is expressed as a percentage.

### Statistical analysis

The data were analyzed using the SPSS v20 package and plotted as the mean ± SEM. Statistical analysis was performed using one-way ANOVA (groups: adult, old AL and old CR) and a Bonferroni correction was applied for multiple comparisons. In addition, Spearman Rank correlations were established to examine the relationship between the hormonal and biochemical variables. A P-value of ≤ 0.05 was considered to be statistically significant.

## Supplementary information

Supplementary Figure 1.
