# Supplementary material for: Caloric restriction modulates the monoaminergic system and metabolic hormones in aged rats
Source: Sci Rep. 2020 Nov 9;10:19299. doi: 10.1038/s41598-020-76219-7 (PMC7653031; doi:10.1038/s41598-020-76219-7)
Supplement: Supplementary file 1 — Supplementary Figure 1. [file 41598_2020_76219_MOESM1_ESM.docx]

**Caloric restriction modulates the monoaminergic system and metabolic hormones in aged rats**

Portero-Tresserra, M*; Rojic-Becker, D; Vega-Carbajal, C; Guillazo-Blanch, G.; Vale-Martínez, A; Martí-Nicolovius, M.

Author Affiliations:

Departament de Psicobiologia i Metodologia de les Ciències de la Salut, Institut de Neurociències, Universitat Autònoma de Barcelona, Barcelona (Spain).

Supplementary information


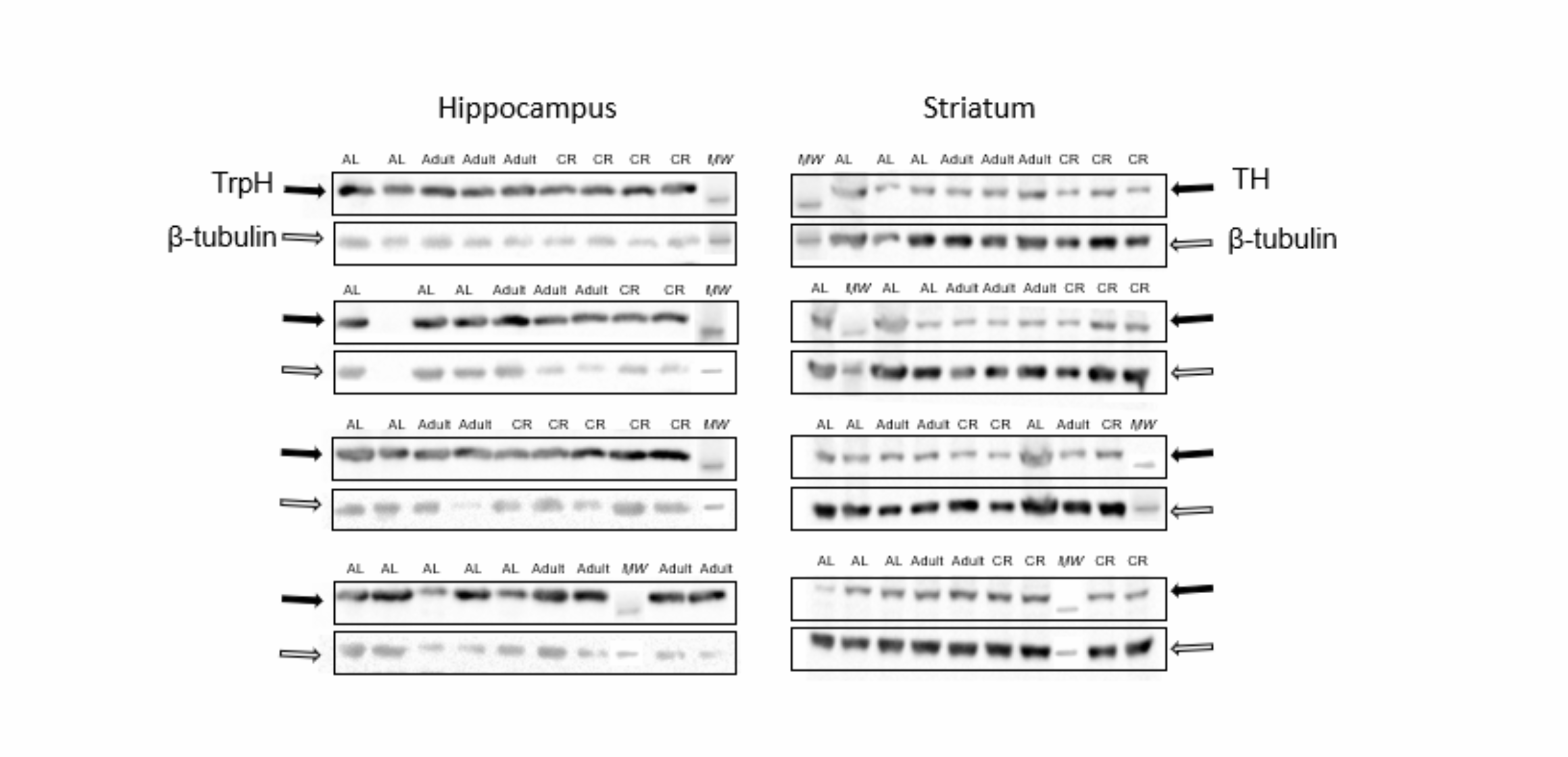


Figure 1. Representative Western Blot analysis for Ad Libitum (AL), Adult and Caloric Restricted (CR) rats in the region of Hippocampus and Striatum. Molecular weight (MW) band represented the molecular weight band of 50 kDa in comparison to the protein expression. Stripping was carried out in both regions due to the proximity of the molecular weight between TrpH (55-57kDa) and TH (60kDa) to the loading control tubulin (50kDa). Clear delineation of each protein is represented with the black line, in order to notice the different moments of exposition and proteins.
